# Postoperative Symptoms and Patient Satisfaction After Standard or Minimally Invasive Pterional Craniotomy for Intracranial Aneurysms

**DOI:** 10.7759/cureus.111828

**Published:** 2026-06-30

**Authors:** Olufunmilola Adeleye, Rohin Singh, Mark A Pacult, Lea Scherschinski, Ethan A Winkler, Mackenzie A Steinbach, Visish M Srinivasan, Joshua S Catapano, John E Wanebo

**Affiliations:** 1 Mayo Clinic Alix School of Medicine, Mayo Clinic, Scottsdale, USA; 2 Department of Neurosurgery, Barrow Neurological Institute, Phoenix, USA; 3 Department of Neurosurgery, HonorHealth Research Institute, Phoenix, USA; 4 Department of Neurosurgery, The John Shufeldt School of Medicine and Medical Engineering, Arizona State University, Phoenix, USA

**Keywords:** anterior circulation aneurysms, frontotemporal sphenoidal craniotomy, intracranial aneurysms, middle cerebral artery aneurysms, minipterional craniotomy

## Abstract

Objective: Microsurgical approaches for intracranial aneurysms are more invasive than endovascular treatments and are associated with longer recovery periods and worse cosmetic outcomes. To reduce the significant surgical burden on patients, neurosurgeons have developed more refined techniques. In this study, headache rates and postoperative scar satisfaction were compared between standard pterional and minipterional craniotomies for anterior circulation aneurysm clipping.

Methods: A single-institution, mixed prospective and retrospective cohort study of patients treated by a single surgeon from 2018 to 2021 was conducted. Headache rate and severity were preoperatively and postoperatively measured with the Migraine Disability Assessment Test (MIDAS) and the Headache Impact Test (HIT-6). Secondary outcomes included cosmetic outcomes, assessed by the SCAR-Q questionnaire, and health function, measured by the 36-Item Short-Form Health Survey (SF-36) and the Patient Global Impression of Change (PGIC).

Results: Of the 36 patients enrolled (25 (69%) women, 11 (31%) men; mean age: 64.9 (range: 34-82) years), 21 underwent standard pterional craniotomy, and 15 underwent minipterional craniotomy. No significant difference was found in HIT-6 and MIDAS grade between approaches; however, a larger percentage of patients in the standard pterional group than in the minipterional group reported severe disability three months postoperatively. All standard-approach patients reported a moderate change in PGIC, but patients in the minipterional group reported the greatest improvement in clinical status. Both groups had similar postoperative headache rates. Patients in the minipterional group had significantly better outcomes for scar appearance than patients in the standard pterional group (p=0.03).

Conclusion: The minipterional approach offered cosmetic advantages without worsening headache burden. Although the sample size and follow-up rates were limited, the results support this less invasive approach as a patient-centered alternative. This work highlights the importance of tailored treatment for brain lesions and offers insight into ways to achieve optimal patient outcomes.

## Introduction

Although endovascular treatment is favored for intracranial aneurysms [1], microsurgery is an excellent option for select lesions. Specifically, it has been proposed that the surgical treatment of anterior circulation aneurysms is associated with superior outcomes because of their unique anatomy and microarchitecture [2,3].

Microsurgical approaches are more invasive than endovascular approaches, with microsurgery resulting in longer recovery periods and less desirable cosmetic outcomes than endovascular surgery [4,5]. Furthermore, surgical disruption of the temporalis muscle poses a risk for postcraniotomy headaches [6]. Smaller, more refined exposures have been developed to ameliorate these drawbacks and minimize the surgical burden on patients. One such approach has been the minimally invasive pterional (i.e., minipterional) craniotomy [7]. A standard pterional or frontotemporal sphenoidal craniotomy involves a 25-cm incision from the ear to the middle of the hairline, whereas a minipterional craniotomy requires an incision of less than a third of that length [8]. The minipterional craniotomy allows adequate exposure and visualization while limiting the incision length [9].

This study aimed to compare headache rates and postoperative scar satisfaction between patients who underwent a minipterional craniotomy and those who underwent a standard craniotomy for microsurgical clipping of middle cerebral artery (MCA) or other anterior circulation aneurysms.

## Materials and methods

Study design and selection criteria

We conducted a single-institution mixed prospective and retrospective case series of patients who underwent surgery for aneurysm clipping by a single surgeon (J.E.W.) between January 2018 and March 2021. We included patients 18 years or older with a diagnosis of MCA aneurysms or other anterior circulation aneurysms who underwent microsurgical clipping via a standard pterional craniotomy or a minimally invasive minipterional craniotomy. The approach was chosen at the discretion of the surgeon intraoperatively based on the aneurysm location, projection, and surgical accessibility. All participants provided informed consent to participate in the study. Patients were excluded if they were under the age of 18 years, had aneurysms located outside the anterior circulation, underwent surgical approaches other than the standard or minipterional craniotomy, or were unable or unwilling to provide informed consent. This study was approved by the HonorHealth Institutional Review Board (IRB# 1471259-1), and patient consent for inclusion in this publication was obtained before data collection. The researchers followed the principles of the 1964 Declaration of Helsinki, and this study has been reported in line with the PROCESS reporting guideline [10]. No trial was registered because the study did not meet the criteria for an applicable clinical trial (42CFR, Part 11).

Surgical technique

The standard pterional craniotomy consisted of a frontotemporal sphenoidal craniotomy via a curvilinear incision about 25 cm in length extending from the tragus to the midline hairline. The craniotomy extended to the sphenoid wing and allowed wide exposure of the sylvian fissure and basal cisterns.

**Figure 1:**
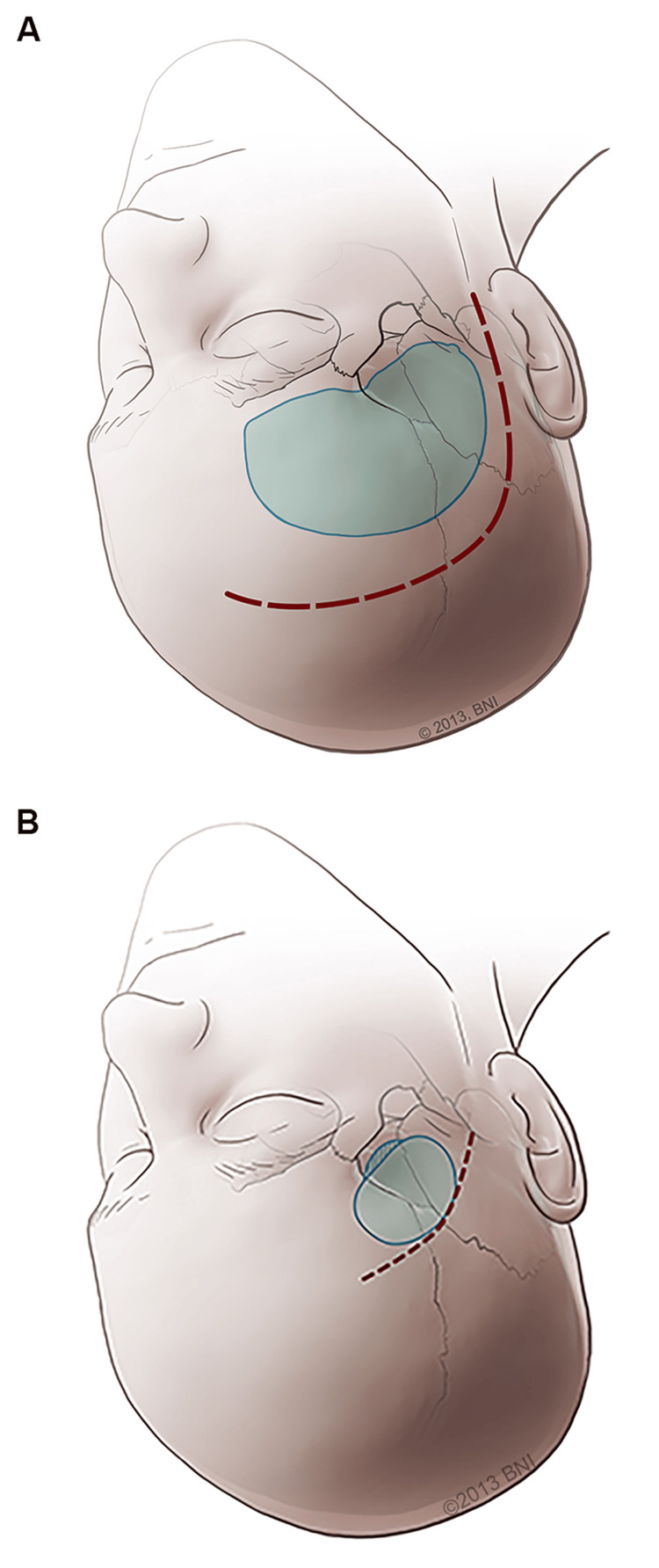
(A) Illustration of a standard frontotemporal pterional craniotomy. (B) Illustration of a minipterional craniotomy. Comparison of the relative size and positioning of the curvilinear scalp incisions shows that those used for a standard frontotemporal craniotomy are longer, more posterior, and have a superior trajectory, whereas those used for a minipterional craniotomy are shorter, more anterior, and have an inferior trajectory. *Used with permission from Barrow Neurological Institute, Phoenix, Arizona.*

The minipterional craniotomy consisted of a smaller curvilinear incision about 6-8 cm in length, placed more anteriorly and inferiorly than the standard pterional craniotomy. Bone removal was limited to the anterior temporal and frontal squama adjacent to the sphenoid ridge, with subfrontal and sylvian fissure access (Figure 1).

Study metrics

Demographic information on patient age, sex, and ethnicity was collected. The primary outcomes of interest were the rate and severity of headaches; these variables were measured with the Migraine Disability Assessment Test (MIDAS) and the Headache Impact Test (HIT-6) [11,12]. The MIDAS assessment consists of five questions, with each answer ranging from 0 to 90 days, which is then rescored into a MIDAS grade; the MIDAS assessment quantifies the degree of impairment experienced by individuals at home, school, or work as a result of headaches (e.g., “On how many days in the last three months did you miss work or school because of your headaches?”). Higher scores reflect a greater number of days with disability and a higher corresponding grade (Table 1).

**Table 1:** Migraine Disability Assessment Test (MIDAS) disability grades corresponding to MIDAS scores.

MIDAS grade	Definition	MIDAS score
I	Little or no disability	0–5
II	Mild disability	6–10
III	Moderate disability	11–20
IV	Severe disability	≥21

The HIT-6 is also a questionnaire with possible scores ranging from 36 to 78 and similarly assesses the effect headaches have on everyday life (e.g., “How often do headaches limit your ability to do usual daily activities, including household work, work, school, or social activities?”). A higher score on the HIT-6 reflects a greater impact from the headaches. Each item is scored using a frequency scale and assigned an item category weight (6, 8, or 12). In this study, headache severity was graded based on the HIT-6 interpretation scoring guide as follows: little or no impact (≤49), some impact (50-55), substantial impact (56-59), and severe impact (60-78).

The secondary outcomes of interest included cosmetic outcomes and general wellness. Cosmetic outcomes were assessed using the SCAR-Q, a questionnaire with three scales that measure the appearance of postsurgical scars (i.e., length, width, color), adverse symptoms based on how the scar feels (i.e., sore, painful, tight, itchy, tingly), and the psychosocial impact and distress caused by scars [13]. A lower score on the SCAR-Q reflects a better outcome. When possible, we collected data using the 36-item Short Form Health Survey (SF-36) to assess patient-reported health evaluations [14]. The SF-36 includes questions regarding eight health dimensions: vitality, physical functioning, bodily pain, general health perceptions, physical role functioning, emotional role functioning, social role functioning, and mental health. A lower score on the SF-36 indicates greater disability; a higher score indicates less disability. The Patient Global Impression of Change (PGIC) scale was also used to assess changes in patients’ perceptions of changes after surgery (i.e., “no change” or “feeling a great deal better”) [15]. Data were collected before the surgery and again at six weeks, three months, six months, and one year postoperatively.

Statistical analysis

This study was based on prospective data and analyzed all available data. Descriptive statistics are presented as mean and standard deviation (SD), frequency, or number and percentage. After confirming that variances were equal between groups using Levene’s test, we used independent t tests to compare continuous variables and Fisher’s exact test to compare categorical variables between groups. All tests were two-tailed, and a p-value <0.05 was considered to represent statistical significance. All analyses were performed using the BlueSky Statistics graphic user interface (version 7.40, BlueSky Statistics, Chicago, IL) for R.

## Results

During the study period, 36 patients underwent microsurgical clipping of MCA or other anterior circulation aneurysms (Table 2).

**Table 2:** Demographic characteristics of patients who underwent standard pterional craniotomy and minipterional craniotomy between January 2018 and March 2021. NR, not reported. Values where n < 11 are not reported to protect patient privacy, except for sex.

Variable	Standard pterional craniotomy (n = 21)	Minipterional craniotomy (n = 15)	Total (N = 36)
Women, n (%)	13 (62)	12 (80)	25 (69)
Men, n (%)	8 (38)	3 (20)	11 (31)
Age, years, mean (SD)	62.4 ± 11.6	68.5 ± 5.7	64.9 ± 1.7
Race, n (%)			
White	19 (90)	12 (80)	31 (86)
Black	NR	NR	NR
Asian	NR	NR	NR
Native American	NR	NR	NR

Twenty-five patients (69%) were women, 11 (31%) were men, and the mean (SD) age was 64.9 (9.9) years (range: 34-82 years). Overall, 21 (58%) patients underwent standard pterional craniotomy, and 15 (42%) underwent minipterional craniotomy. There were no significant differences in patients’ age, sex, or ethnicity between groups. Operative metrics were not available for analysis.

An independent-sample t test was conducted to compare primary and secondary outcomes after standard pterional craniotomy and minipterional craniotomy. No significant difference was found between the mean (SD) MIDAS grade for standard pterional craniotomy (90 (127.28)) and the minipterional craniotomy (38.57 (102.05)) after a three-month follow-up (Table 3).

**Table 3:** Outcomes after standard pterional craniotomy or minipterional craniotomy in patients undergoing middle cerebral artery and other anterior circulation aneurysm clipping between January 2018 and March 2021* HIT-6, Headache Impact Test; MIDAS, Migraine Disability Assessment Test; SF-36, 36-Item Short-Form Health Survey. *All values are expressed as mean ± standard deviation. †Bold type indicates statistical significance (p < 0.05).

Assessment, follow-up	Standard pterional craniotomy (n = 21)	Minipterional craniotomy (n = 15)	p-Value^†^
Primary Outcomes			
MIDAS Score			
Baseline	4 ± 0.00 (n = 1)	2.2 ± 4.84 (n = 9)	0.73
Six weeks	31.6 ± 54.84 (n = 3)	32.42 ± 63.69 (n = 7)	0.98
Three months	90 ± 127.28 (n = 2)	38.57 ± 102.05 (n = 7)	0.56
HIT-6			
Baseline	49 ± 18.30 (n = 2)	47.1 ± 8.00 (n = 10)	0.80
Six weeks	48.66 ± 11.15 (n = 3)	44.5 ± 12.25 (n = 9)	0.61
Three months	48 ± 11.88 (n = 7)	44.5 ± 12.48 (n = 10)	0.57
Six months	41.28 ± 7.08 (n = 7)	43.85 ± 13.4 (n = 7)	0.66
One year	49 ± 14.13 (n = 8)	44.7 ± 10.1 (n = 4)	0.61
Secondary Outcomes			
SCAR-Q Appearance			
Baseline	13.5 ± 0.70 (n = 2)	16.3 ± 5.46 (n = 11)	0.48
Six weeks	12.3 ± 0.57(n = 3)	14.33 ± 2.69 (n = 9)	0.24
Three months	12.5 ± 0.97 (n = 7)	12.4 ± 1.26 (n = 10)	0.76
Six months	15.2 ± 3.45 (n = 7)	12.14 ± 0.37 (n = 7)	0.03
One year	16.3 ± 4.30 (n = 8)	12 ± 0.00 (n = 4)	0.07
SCAR-Q Symptoms			
Baseline	17 ± 0.00 (n = 1)	15.5 ± 4.33 (n = 9)	0.75
Six weeks	20 ± 6.43 (n = 3)	16.7 ± 2.75 (n = 7)	0.19
Three months	18 ± 5.29 (n = 3)	14.57 ± 3.64 (n = 7)	0.20
SCAR-Q Psychosocial Impact			
Baseline	5 ± 0.00 (n = 1)	5.6 ± 1.30 (n = 9)	0.64
Six weeks	5.33 ± 0.33 (n = 3)	5 ± 0.00 (n = 7)	0.13
Three months	5 ± 0.00 (n = 2)	5.28 ± 0.75 (n = 7)	0.62
SF-36			
Baseline	97 ± 0.00 (n = 1)	100 ± 9.60 (n = 9)	0.76
Six weeks	98.6 ± 7.09 (n = 3)	99 ± 5.940 (n = 7)	0.94
Three months	108 ± 7.77 (n = 2)	101 ± 3.33 (n = 7)	0.09

**Figure 2:**
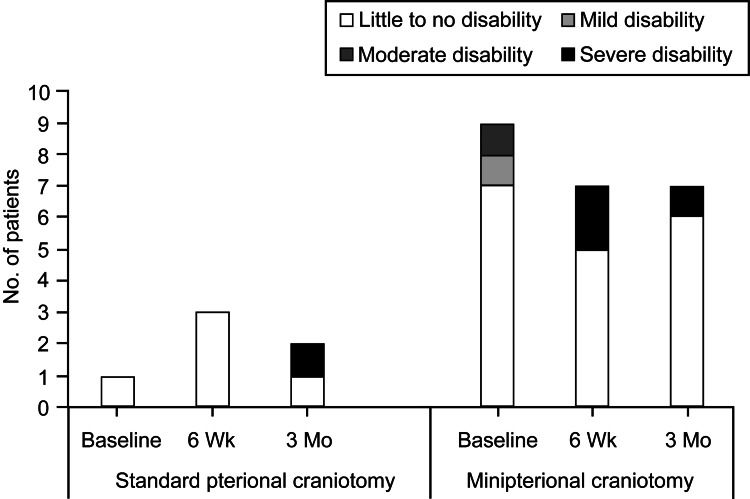
Temporal trends in MIDAS grades based on the surgical approach. Changes in MIDAS grade over time, grouped by type of surgery. MIDAS, Migraine Disability Assessment Test. *Used with permission from Barrow Neurological Institute, Phoenix, Arizona.*

In the standard pterional craniotomy group, one patient completed the questionnaire preoperatively and reported little to no disability. Two patients in the standard pterional craniotomy group completed the questionnaire at the three-month follow-up. One reported little to no disability, and one reported severe disability. In the minipterional craniotomy group, nine patients completed the MIDAS questionnaire preoperatively: seven reported little to no disability, one reported mild disability, and one reported moderate disability. At the three-month follow-up, seven patients in the minipterional craniotomy group completed the questionnaire: six reported little to no disability, and one reported severe disability (Figure 2).

No significant differences in the HIT-6 score were found between the standard pterional craniotomy and minipterional craniotomy groups at three months (48 (11.88) vs 44.5 (12.48)) or one year (49 (14.13) vs 44.7 (10.1)) after the operation.

At the six-month follow-up, a significant difference was found in the SCAR-Q appearance scores between patients who underwent a standard pterional craniotomy and patients who underwent a minipterional craniotomy (15.2 (3.45) vs 12.14 (0.37); t (degrees of freedom (DF)) = 12; p = 0.03). At the one-year follow-up, the difference in the same metric between patients who underwent a standard pterional craniotomy and patients who underwent a minipterional craniotomy were 16.3 (4.3) and 12 (0.0), respectively (mean difference = −4.37, 95% confidence interval (CI), −9.29 to 0.54; t(DF) = 10; p = 0.07). No significant differences were found in the SCAR-Q symptom score between the standard pterional craniotomy and minipterional craniotomy groups at the three-month follow-up (18 (5.29) and 14.57 (3.64), respectively). Similarly, no significant differences were found in SCAR-Q psychosocial score between the standard pterional craniotomy and minipterional craniotomy groups at the three-month follow-up (5 (0.0) vs 5.28 (0.75)). No significant differences were observed in the SF-36 scores between the standard pterional craniotomy and minipterional craniotomy groups (108 (7.7) vs 101 (3.3)) at the three-month follow-up.

**Figure 3:**
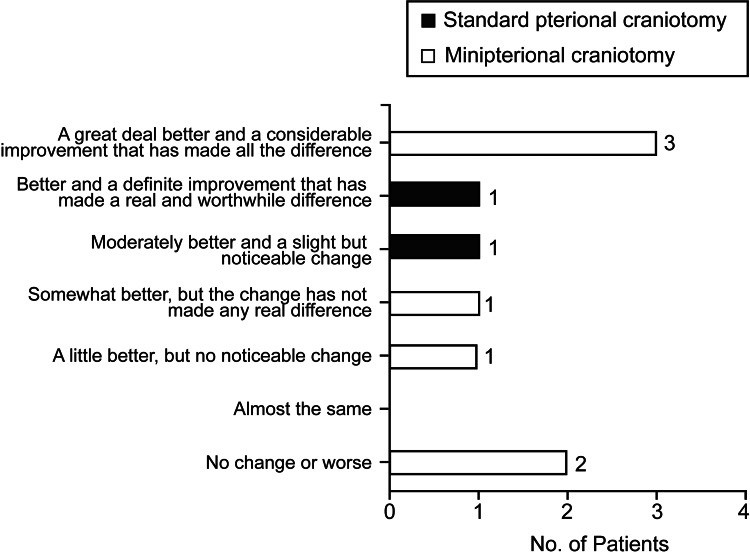
Patient Global Impression of Change (PGIC) responses comparing standard and minipterional craniotomy at the 3-month follow-up. PGIC responses for two patients with standard pterional craniotomy and seven patients with minipterional craniotomy at 3 months after the operation. *Used with permission from Barrow Neurological Institute, Phoenix, Arizona.*

PGIC scores were collected postoperatively. As assessed by the PGIC scale, one of two patients who underwent a standard pterional craniotomy reported feeling “moderately better and a slight but noticeable change,” and one reported feeling “better and a definite improvement that has made a real and worthwhile difference.” In the minipterional group, two reported feeling “no change or worse,” one reported feeling “a little better, but no noticeable change,” one reported feeling “somewhat better, but the change has not made any real difference,” and three reported feeling “a great deal better and a considerable improvement that has made all the difference” (Figure 3).

**Figure 4:**
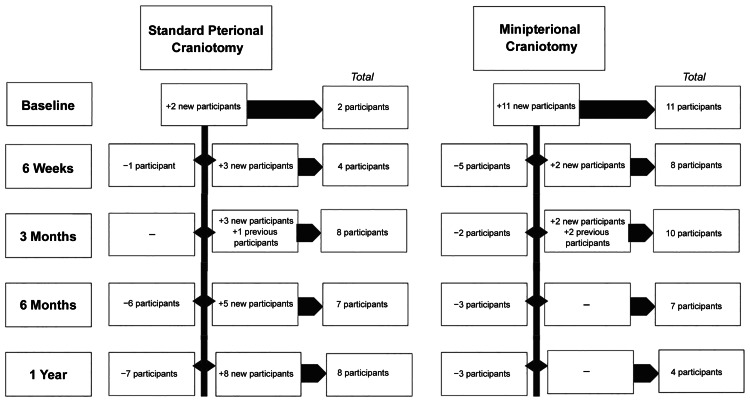
Patient enrollment and study participation for craniotomy approaches. Enrollment and responses for patients undergoing a standard pterional craniotomy versus minipterional craniotomy from January 2018 to March 2021. *Used with permission from Barrow Neurological Institute, Phoenix, Arizona.*

Details on enrollment are provided in Figure 4.

## Discussion

In this study, we analyzed the outcomes of the standard and minipterional craniotomy approaches for MCA and other anterior circulation aneurysms and sought to answer two major questions: Are the safety and efficacy of the newer approach at least equal to the safety and efficacy of the standard approach? If so, does the new approach offer any new benefits to the patient?

This study adds to the growing literature demonstrating the efficacy of the minipterional approach in various neurosurgical procedures, including aneurysm repairs [16-18] and cavernous malformation resections [19,20]. Although we found no statistically significant difference in the HIT-6 score or MIDAS grade between the standard approach and minipterional approaches, a larger percentage of people in the standard-approach group than in the minipterional group reported severe disability 3 months after surgery (Figure 2). Previous studies have reported postoperative headaches in more than 30% of patients who have undergone craniotomy, and headache is thus cited as one of the most common adverse events after neurosurgery [21,22]. When comparing headache rates between the two groups, it was found that the minipterional approach was not associated with increased disability compared with the standard pterional approach. Although the difference was not statistically significant, it might be of clinical significance that fewer patients in the minipterional group reported scores consistent with moderate or severe headache-related disability postoperatively than patients in the standard pterional group.

In this study, patients in the minipterional group had significantly better scar appearance outcomes than patients in the standard pterional group. Park et al. found that the main determinant of patient satisfaction is the cosmetic result, especially for aneurysm surgery [23]. As a quality metric, patient satisfaction is a desired outcome for surgery [24]. The benefit of less postoperative scarring answers the second question and highlights a substantial advantage of the minipterional approach. Interestingly, there was no difference in the other two scales measured by the SCAR-Q (i.e., symptoms and psychosocial impact). The SCAR-Q symptom scale describes the negative characteristics of the scar and the physical symptoms it is associated with. Accordingly, one would expect those symptoms to decrease after recovery. On the other hand, the SCAR-Q psychosocial scale measures the psychological distress attributed to the surgical scar. With patients undergoing craniotomies, the response can be interpreted to indicate that, although the appearance of the scar might cause some dissatisfaction, the seriousness of the procedure outweighs cosmetic concerns. This interpretation coincides with the thoughts of similar patients who underwent breast reconstruction and answered questions under the SCAR-Q psychosocial domain: “The fact that I have two scars that go across... thank God I can have two scars that go across, and I am not six feet under!” [13]. Another study highlighted that an additional benefit of cosmetically functional and less invasive techniques is the appeal to young patients with aneurysms, which are increasingly detected earlier with routine brain imaging [25].

Overall, we found a wide range in the PGIC subjective scores of patients in this study (Figure 3). All patients in the standard pterional group reported that they were moderately better or that an improvement had been made. However, patients in the minipterional group reported the most meaningful change, experiencing the highest level of postoperative improvement in clinical status (i.e., “A great deal better, and a considerable improvement that has made all the difference”). On the other hand, no significant differences were observed in the SF-36, demonstrating that the minipterional approach does not differ from the standard approach in terms of patients’ perceptions of their health.

This study has several potential limitations. First, although this study was prospective, some patients were recruited retrospectively; therefore, we could not collect baseline data for all patients, and participants were recruited at different postoperative time points, making the study underpowered to detect an important effect of the interventions evaluated. Second, not all questionnaires were completed, and some participants were lost to follow-up, leading to incomplete data collection. As with any longitudinal study, the loss of participants to follow-up is typical and resulted in incomplete datasets, particularly for later timepoints; we believe capturing time-to-event data offers standardization and helps minimize bias. Third, the patients were treated by a single surgeon at a single institution, limiting the generalizability of our findings. Treatment assignment was nonrandomized and based on aneurysmal characteristics. Additionally, operative details and postoperative details, including complications and skin incision images, were not consistently recorded and were excluded. Lastly, there may be a response bias because the results are self-reported on standardized questionnaires. These limitations reduce statistical power and generalizability but are transparently acknowledged. To our knowledge, this study is the first to compare long-term disability and cosmetic outcomes between a conventional cranial approach and a minimally invasive approach in the United States, which makes these findings an important addition to the literature.

Future studies could expand on this work by evaluating preoperative patient and provider goals and examining how they align with postoperative outcomes for the two approaches. This series does not attempt to replace the standard pterional approach. However, the minipterional approach offers options that may prove beneficial in surgical decision-making, depending on the neurosurgeon’s expertise.

## Conclusions

In craniotomies performed to repair intracranial aneurysms, the standard pterional and minipterional approaches offer comparable outcomes, with minimal disability caused by headaches. However, the minipterional approach is less invasive and offers better cosmetic results than the standard pterional approach, thereby increasing patient satisfaction.

## References

[1] Sharma M, Ugiliweneza B, Fortuny EM (2019). National trends in cerebral bypass for unruptured intracranial aneurysms: a National (Nationwide) Inpatient Sample analysis of 1998-2015. Neurosurg Focus.

[2] Darsaut TE, Keough MB, Sagga A (2021). Surgical or endovascular management of middle cerebral artery aneurysms: a randomized comparison. World Neurosurg.

[3] Rodriguez-Hernandez A, Sughrue ME, Akhavan S, Habdank-Kolaczkowski J, Lawton MT (2013). Current management of middle cerebral artery aneurysms: surgical results with a "clip first" policy. Neurosurgery.

[4] Hoh BL, Chi YY, Dermott MA, Lipori PJ, Lewis SB (2009). The effect of coiling versus clipping of ruptured and unruptured cerebral aneurysms on length of stay, hospital cost, hospital reimbursement, and surgeon reimbursement at the university of Florida. Neurosurgery.

[5] Hoh BL, Chi YY, Lawson MF, Mocco J, Barker FG, 2nd 2nd (2010). Length of stay and total hospital charges of clipping versus coiling for ruptured and unruptured adult cerebral aneurysms in the Nationwide Inpatient Sample database 2002 to 2006. Stroke.

[6] Brazoloto TM, de Siqueira SR, Rocha-Filho PA, Figueiredo EG, Teixeira MJ, de Siqueira JT (2017). Post-operative orofacial pain, temporomandibular dysfunction and trigeminal sensitivity after recent pterional craniotomy: preliminary study. Acta Neurochir (Wien.

[7] Yagmurlu K, Safavi-Abbasi S, Belykh E (2017). Quantitative anatomical analysis and clinical experience with mini-pterional and mini-orbitozygomatic approaches for intracranial aneurysm surgery. J Neurosurg.

[8] Chaddad-Neto F, Campos Filho JM, Doria-Netto HL, Faria MH, Ribas GC, Oliveira E (2012). The pterional craniotomy: tips and tricks. Arq Neuropsiquiatr.

[9] Gandhi S, Cavallo C, Zhao X (2018). Minimally invasive approaches to aneurysms of the anterior circulation: selection criteria and clinical outcomes. J Neurosurg Sci.

[10] Agha RA, Sohrabi C, Mathew G (2020). The PROCESS 2020 Guideline: updating consensus Preferred Reporting Of CasESeries in Surgery (PROCESS) guidelines. Int J Surg.

[11] Stewart WF, Lipton RB, Dowson AJ, Sawyer J (2001). Development and testing of the Migraine Disability Assessment (MIDAS) Questionnaire to assess headache-related disability. Neurology.

[12] Kosinski M, Bayliss MS, Bjorner JB (2003). A six-item short-form survey for measuring headache impact: the HIT-6. Qual Life Res.

[13] Klassen AF, Ziolkowski N, Mundy LR (2018). Development of a new patient-reported outcome instrument to evaluate treatments for Scars: The SCAR-Q. Plastic and reconstructive surgery Global open.

[14] Ware JE, Jr. Jr., Snow KK, Kosinski M, Gandek B (1993). SF-36 Health survey: manual and interpretation guide. https://www.researchgate.net/profile/John-Ware-6/publication/313050850_SF-36_Health_Survey_Manual_Interpretation_Guide/links/594a5b83aca2723195de5c3d/SF-36-Health-Survey-Manual-Interpretation-Guide.pdf.

[15] Dworkin RH, Turk DC, Wyrwich KW (2008). Interpreting the clinical importance of treatment outcomes in chronic pain clinical trials: IMMPACT recommendations. J Pain.

[16] Davies JM, Lawton MT (2014). Advances in open microsurgery for cerebral aneurysms. Neurosurgery.

[17] Park JS, Kwon MY, Lee CY (2020). Minipterional craniotomy for surgical clipping of anterior circulation aneurysms: compatibility between the feasibility, safety and efficiency. J Cerebrovasc Endovasc Neurosurg.

[18] Di Bonaventura R, Sturiale CL, Latour K, Mazzucchi E, Marchese E, Albanese A (2021). Comparison between minipterional craniotomy associated with focused sylvian fissure opening and standard pterional approach with extended sylvian fissure dissection for treatment of unruptured middle cerebral artery aneurysms. World Neurosurg.

[19] Singh R, Srinivasan VM, Lawton MT (2021). Minipterional transsylvian approach for resection of a cavernous malformation in the optic chiasm. World Neurosurg.

[20] Martinez-Perez R, Hernandez-Alvarez V, Maturana R, Mura JM (2019). The extradural minipterional pretemporal approach for the treatment of spheno-petro-clival meningiomas. Acta Neurochir (Wien.

[21] Tullos HJ, Conner AK, Baker CM (2018). Mini-pterional craniotomy for resection of parasellar meningiomas. World Neurosurg.

[22] Subbarao BS, Fernandez-de Thomas RJ, Das JM, Eapen BC (2025). Postcraniotomy Headache. https://www.ncbi.nlm.nih.gov/books/NBK482297.

[23] Park J, Son W, Kwak Y, Ohk B (2019). Pterional versus superciliary keyhole approach: direct comparison of approach-related complaints and satisfaction in the same patient. J Neurosurg.

[24] Sacks GD, Lawson EH, Dawes AJ (2015). Relationship between hospital performance on a patient satisfaction survey and surgical quality. JAMA Surg.

[25] Kim E, Delashaw JB, Jr Jr (2011). Osteoplastic pterional craniotomy revisited. Neurosurgery.

